# P-1551. Prevalence and Risk Factors of Multidrug Resistance in Patients with Complicated Urinary Tract Infections (cUTIs) and all UTIs: A Systematic Literature Review

**DOI:** 10.1093/ofid/ofae631.1718

**Published:** 2025-01-29

**Authors:** Fanny S Mitrani-Gold, Myriam Drysdale, Saifuddin Kharawala, Pooja Malhotra, Alanna Farrell-Foster, Jeffrey J Ellis, Emily Lloyd

**Affiliations:** GlaxoSmithKline plc., Collegeville, Pennsylvania; GSK, Brentford, Middlesex, England, United Kingdom; Bridge Medical Consulting Limited, London, England, United Kingdom; Bridge Medical Consulting Limited, London, England, United Kingdom; GSK, Brentford, Middlesex, England, United Kingdom; GSK, Brentford, Middlesex, England, United Kingdom

## Abstract

**Background:**

Gram-negative pathogens frequently implicated in complicated urinary tract infections (cUTIs) include Enterobacterales (*Escherichia coli* [*E. coli*], *Klebsiella* and *Proteus* species). These can harbor multidrug resistance mechanisms such as extended-spectrum β-lactamases (ESBLs) limiting the effectiveness of first-line antibiotics. We studied the prevalence of, and risk factors for, multidrug resistant (MDR) infections among patients with cUTI and for all UTIs.

**Figure:**
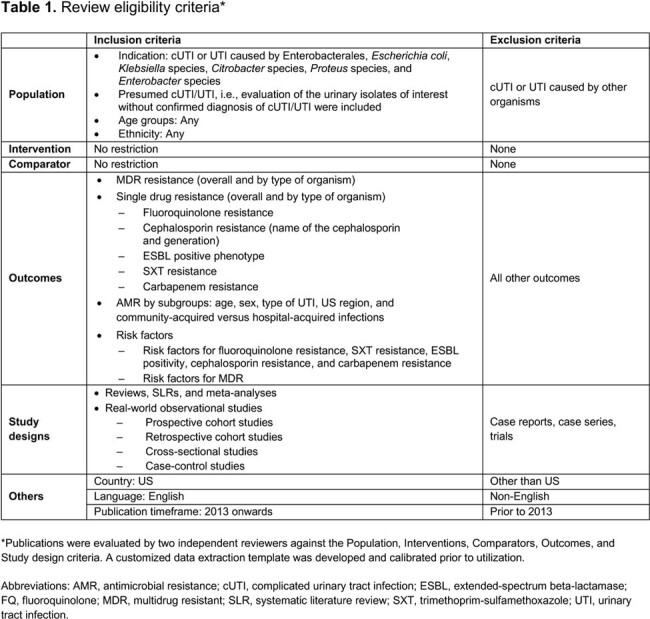

**Methods:**

Systematic searches were conducted in MEDLINE and Embase, supplemented by conference, bibliography, and keyword-based searches (2013–2024). All US studies reporting data on antimicrobial resistance (AMR) in either confirmed or presumed cUTI and UTI involving bacterial isolates of interest were included (**Table 1**). A data extraction template was developed a priori and calibrated before use. AMR results were summarized for cUTI and UTIs (uncomplicated UTI [uUTI], mixed uUTI/cUTI, cUTI), as some studies reported a mixture of cUTI and uUTI, or UTI severity not specified.

**Figure:**
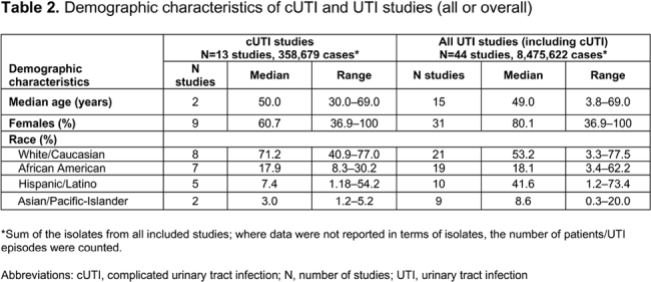

**Results:**

We included 44 US primary studies in UTI (13 cUTI, 31 all UTIs), with results summarized for all studies identified for cUTI and all UTIs. The median age range across studies was 3.8–69.0 years (**Table 2**). Most patients were White/Caucasian (median 53.2%); MDR ≥ 3 was the most commonly reported AMR phenotype. Among cUTI studies, MDR ≥ 3 was seen in 9.9% of *E. coli* and 10.1% of *Klebsiella* spp.; for all UTIs studies, it was 6.9% and 4.6%, respectively. The MDR ≥ 3 proportion for Enterobacterales was 12.5% and 11.3% among cUTI/all UTIs, respectively (**Table 3**). Similar estimates were observed when assessing AMR to individual drug classes with a higher proportion of AMR observed among cUTI isolates vs isolates from all UTIs (**Table 4**). Within individual studies, resistance rates were generally higher in hospital-acquired vs community-acquired infections, in recurrent vs non-recurrent UTI, in catheter-associated vs non-catheter-associated UTI, in cUTI vs uUTI, and in pyelonephritis vs cystitis. AMR was higher among UTI isolates from men and older patients.

**Figure:**
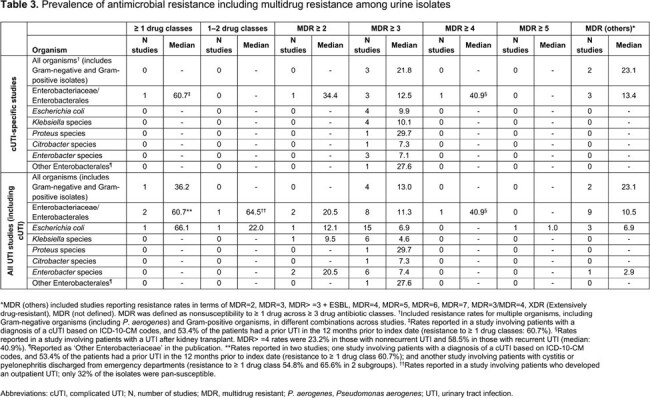

**Conclusion:**

cUTIs had a higher prevalence of MDR uropathogens vs all UTIs. AMR prevalence in cUTI was higher among men, older patients, and patients with recurrent UTI.

**Funding:** GSK study 221807

**Figure:**
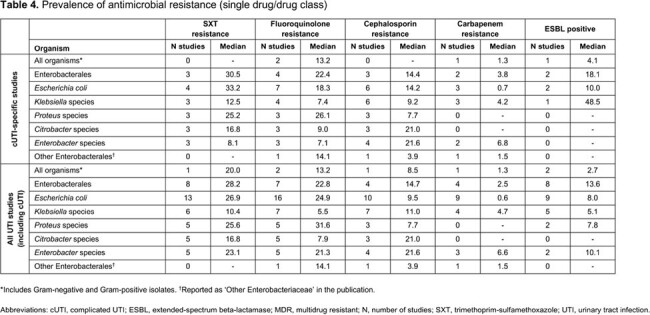

**Disclosures:**

**Fanny S. Mitrani-Gold, MPH**, GSK: Employee|GSK: Stocks/Bonds (Public Company) **Myriam Drysdale, PhD**, GSK: Employee|GSK: Stocks/Bonds (Public Company) **Saifuddin Kharawala, DPM**, Bridge Medical Consulting Ltd.: Employee of Bridge Medical Consulting Ltd., who received funding from GSK to complete this study|GSK India: Previous employee **Pooja Malhotra, MPharm**, Bridge Medical Consulting Ltd.: Employee of Bridge Medical Consulting Ltd., who received funding from GSK to complete this study **Alanna Farrell-Foster**, GSK: Employee|GSK: Stocks/Bonds (Public Company) **Jeffrey J. Ellis, PharmD, MS**, GSK: Employee|GSK: Stocks/Bonds (Public Company) **Emily Lloyd, MSc**, GSK: Employee|GSK: Stocks/Bonds (Public Company)

